# Soluble B7‐H6 as a Novel Diagnostic Biomarker for Post‐Hepatitis B Cirrhosis: A Clinical Value Study

**DOI:** 10.1002/jcla.70288

**Published:** 2026-06-23

**Authors:** Kaiyong Chen, Luxuan Yang, Huaiwu Fu, Lei Cao, Zhu Li

**Affiliations:** ^1^ The Affiliated Guanyun Hospital of Kangda College of Nanjing Medical University Lianyungang Jiangsu China; ^2^ Department of Clinical Laboratory Guanyun People's Hospital Lianyungang Jiangsu China; ^3^ Jiangsu Institute of Clinical Immunology The First Affiliated Hospital of Soochow University Suzhou Jiangsu China; ^4^ Department of Infectious Diseases The Affiliated Infectious Diseases Hospital of Soochow University (The Fifth People's Hospital of Suzhou) Suzhou Jiangsu China; ^5^ Department of Emergency Medicine Guanyun People's Hospital Lianyungang Jiangsu China

**Keywords:** cirrhosis, diagnostic biomarker, hepatitis B, non‐invasive diagnosis, ROC curve, soluble B7‐H6

## Abstract

**Objective:**

This study aimed to assess the diagnostic value of soluble B7‐H6 (sB7‐H6) as a biomarker for post‐hepatitis B cirrhosis (LC) and to compare its performance with conventional non‐invasive biomarkers, including FIB‐4 and APRI.

**Methods:**

A prospective cohort study was conducted with 159 patients diagnosed with HBV‐related liver diseases, including 77 with chronic hepatitis B (CHB) and 82 with post‐hepatitis B cirrhosis (LC). Serum sB7‐H6 levels were quantified using enzyme‐linked immunosorbent assay (ELISA). The diagnostic performance of sB7‐H6 was evaluated through receiver operating characteristic (ROC) curve analysis. The diagnostic accuracy of sB7‐H6, both alone and in combination with FIB‐4, was compared to the traditional biomarkers APRI and FIB‐4.

**Results:**

Serum sB7‐H6 levels increased progressively with disease severity, with the highest levels observed in the LC group. The area under the ROC curve (AUC) for sB7‐H6 alone in diagnosing cirrhosis was 0.87, which was superior to both APRI (AUC = 0.75) and FIB‐4 (AUC = 0.79). The combined model of sB7‐H6 and FIB‐4 demonstrated improved diagnostic performance, achieving an AUC of 0.92, with sensitivity of 85.4% and specificity of 88.0%.

**Conclusion:**

Serum sB7‐H6 is a promising, non‐invasive biomarker for diagnosing post‐hepatitis B cirrhosis, outperforming traditional biomarkers such as FIB‐4 and APRI. The combined model of sB7‐H6 and FIB‐4 provides superior diagnostic accuracy and holds significant potential for clinical use in the early detection and risk stratification of liver cirrhosis in HBV‐infected patients.

## Graphical abstract

This study investigates the potential of soluble B7‐H6 (sB7‐H6) as a novel diagnostic biomarker for post‐hepatitis B cirrhosis. The infographic visually presents the following key points: (1) *Liver Disease Progression*: Chronic hepatitis B (CHB) progresses to liver cirrhosis (LC), with serum sB7‐H6 levels increasing stepwise as the disease advances. (2) *Comparison of Biomarkers*: sB7‐H6 is shown to outperform traditional biomarkers such as FIB‐4 and APRI in diagnosing cirrhosis, as demonstrated by its higher diagnostic performance. (3) *Diagnostic Performance*: An ROC curve comparison highlights that sB7‐H6, when combined with FIB‐4, achieves superior diagnostic accuracy (AUC = 0.92), with high sensitivity and specificity. (4) *Clinical Impact*: sB7‐H6 provides a non‐invasive, high AUC diagnostic tool for accurately identifying liver cirrhosis, offering a valuable option for clinical practice.

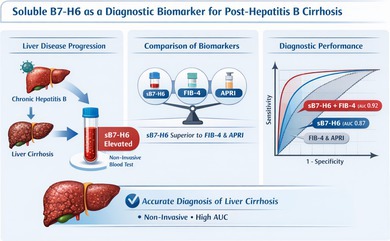

## 1 Introduction

Chronic hepatitis B virus (HBV) infection is a major global public health concern. According to the World Health Organization, approximately 296 million people worldwide are chronically infected with HBV, and around 820,000 people die annually from HBV‐related end‐stage liver disease [1]. China bears the heaviest burden of HBV infection, with over 86 million individuals currently infected. Additionally, 84% of hepatocellular carcinoma (HCC) cases are closely associated with chronic HBV infection [2, 3]. Notably, the annual incidence of cirrhosis progression among individuals with chronic HBV infection can reach 2%–10%, and the annual risk of developing HCC in cirrhosis patients is as high as 3%–6% [4].

The early pathological process of cirrhosis is often insidious and reversible. However, once the disease progresses to decompensated cirrhosis, the 5‐year survival rate significantly declines to less than 50% [5]. Therefore, early detection of cirrhosis and timely intervention are critical to improving patient prognosis. While liver biopsy remains the gold standard for diagnosing liver fibrosis and cirrhosis, its invasive nature, which carries risks such as bleeding, pain, and potential sampling errors, limits its widespread clinical application [6].

In recent years, non‐invasive diagnostic technologies have made significant advances. Transient elastography (TE), which measures liver stiffness (LSM), is commonly used to assess the degree of fibrosis. However, its results can be influenced by factors such as obesity and liver inflammation [7]. Additionally, serological markers like APRI and FIB‐4 are recommended by multiple guidelines for evaluating liver fibrosis. However, these models were originally developed using data from HCV‐infected individuals, making their diagnostic accuracy limited in HBV‐infected populations [8, 9]. Several studies have shown that APRI and FIB‐4 are less effective for diagnosing HBV‐related cirrhosis, highlighting the need for the development of new serum biomarkers.

The liver damage mechanism following HBV infection is primarily associated with the host immune response rather than the direct effects of the virus [4]. Immune checkpoint molecules play a crucial role in immune regulation networks, fine‐tuning the intensity of immune responses through co‐stimulatory and co‐inhibitory signals [10]. Abnormal expression of immune checkpoint molecules during chronic HBV infection contributes to disease progression [11, 12, 13, 14, 15]. These molecules form soluble forms (soluble immune checkpoints, sIC) through membrane shedding or selective splicing and maintain biological activity in peripheral blood, making them ideal disease diagnostic biomarkers [16, 17, 18].

B7‐H6 is a new member of the B7 family and serves as a ligand for the NK cell activation receptor NKp30. It is normally expressed at low levels in healthy tissues but is significantly upregulated in tumor and inflammatory conditions [19, 20, 21, 22, 23]. The soluble form, sB7‐H6, is generated through extracellular domain shedding mediated by ADAM proteases [24]. In malignant tumors such as pancreatic cancer and cervical cancer, sB7‐H6 has demonstrated diagnostic value [25, 26]. Studies have shown that B7‐H6 expression is significantly higher in liver cells during damage or carcinogenesis compared to normal tissues [27, 28]. However, there is currently a lack of systematic research on the dynamic changes and diagnostic value of sB7‐H6 in the progression of HBV‐related liver disease.

This study is the first to systematically investigate the expression profile and diagnostic value of sB7‐H6 in HBV‐related liver disease. Through clinical sample analysis, we explored the correlation between sB7‐H6 and disease progression, liver injury markers, and fibrosis biomarkers. We further assessed its diagnostic potential for post‐hepatitis B cirrhosis, aiming to provide a new serum biomarker for non‐invasive diagnosis of post‐hepatitis B cirrhosis.

## 2 Materials and Methods

### 2.1 Study Participants and Grouping

This study employed a prospective cohort design, enrolling 159 patients with HBV‐related liver diseases who were treated at the Department of Hepatology, The First Affiliated Hospital of Soochow University, between October 2022 and October 2023. The cohort included 77 patients with chronic hepatitis B (CHB) and 82 patients with post‐hepatitis B cirrhosis (LC). All eligible patients were enrolled continuously to minimize selection bias.

- *Inclusion Criteria*: All CHB and LC patients underwent ultrasound‐guided liver biopsy. The histopathological diagnosis of liver tissue was made independently by two or more senior pathologists, who were blinded to the clinical data.
- *CHB Group*: Liver histology showed inflammation grading ≥ G2 and/or fibrosis staging ≥ S2 but did not meet the criteria for cirrhosis (S4).
- *LC Group*: Liver histology confirmed cirrhosis (METAVIR score F4 or Ishak score 5–6).
- *Exclusion Criteria*: ① Presence of other liver diseases (e.g., other viral hepatitis, alcoholic liver disease, etc.); ② History of malignancy; ③ Recent severe infection; ④ Autoimmune diseases; ⑤ Pregnancy or breastfeeding.

Additionally, 42 healthy individuals were selected as the healthy control (HC) group. The HC group was matched for age and gender to ensure comparability with the CHB and LC groups. Specifically, all participants were between 18 and 60 years of age. The sex ratio and age range in the HC group were similar to those of the HBV patient groups, ensuring sample balance and the validity of the study results.

During the screening process, all HC participants were confirmed to be free from chronic hepatitis B, other liver diseases, liver function abnormalities, a history of malignancy, or immune system‐related disorders. These strict exclusion criteria ensured that the HC group was healthy and provided an accurate comparison with the liver disease groups.

### 2.2 Sample Collection and Processing

Venous blood samples were collected from all participants in the morning after overnight fasting. A 2‐mL sample of whole blood was collected in an EDTA‐K2 anticoagulant tube for routine blood tests, and 5 mL of venous blood was collected in a serum separation tube containing a coagulation activator. After standing at room temperature for 30 min, blood samples were centrifuged at 500 × *g* for 10 min to separate the serum. The serum was then stored at −80°C until further analysis. Serum samples and liver biopsies were collected on the same day or within 1 week to avoid interference from disease progression on the results.

### 2.3 Experimental Methods

#### 2.3.1 Detection of sB7‐H6

Serum sB7‐H6 levels were quantified using a double‐antibody sandwich enzyme‐linked immunosorbent assay (ELISA), with the kit purchased from Suzhou Xuguang Kexing Biotechnology Co. Ltd. All samples were processed in the same batch to reduce inter‐batch variation. The procedure followed the manufacturer's instructions and was summarized as follows:

- 100 μL of the standard and serum samples were added to the reaction wells and incubated at 37°C for 2 h;
- After washing 3 times, 100 μL of horseradish peroxidase‐labeled primary antibody working solution was added, followed by incubation at 37°C for 1 h:
- After a second wash, 100 μL of enzyme‐labeled secondary antibody was added and incubated at 37°C for 1 h;
- After washing 6 times, 100 μL of TMB substrate was added to each well, and the wells were incubated at room temperature in the dark for 15 min;
- 50 μL of stop solution was added to terminate the reaction, and absorbance was measured at 450 nm using a microplate reader within 10 min. A standard curve was plotted based on the concentrations of standards and the absorbance values to calculate the sB7‐H6 concentration in the samples, expressed in pg/mL.

#### 2.3.2 Other Laboratory Measurements

- *Liver Fibrosis Markers*: HA, LN, PIII NP, IV‐C, and HBV serological markers (HBsAg, HBeAg) were measured using an automatic chemiluminescence immunoassay analyzer (A2000Plus, AnTu, Zhengzhou) and its corresponding reagents.
- *Liver Function Tests*: ALT, AST, and TBil were measured using an automatic biochemical analyzer (FX8, Canon, Japan), with reagents purchased from Beijing AnTu Bioengineering Co. Ltd.
- *HBV‐DNA Quantification*: HBV‐DNA levels were quantified using fluorescence quantitative PCR (LineGene 9600 Plus, Biorex, Hangzhou), with reagents purchased from Guangzhou Kaipu Biotech Co. Ltd.
- *Inflammatory Cytokine IL‐6*: Measured by ELISA using kits purchased from Shanghai Enzyme‐Linked Biotech Co. Ltd., following the same protocol as for sB7‐H6.
- *Complete Blood Count*: Performed using an automated blood cell analyzer (BC‐7500, Mindray, Shenzhen).

#### 2.3.3 Calculation of Non‐Invasive Diagnostic Models

Non‐invasive diagnostic models were calculated based on laboratory test results [4]:

$$\begin{array}{c} \text{APRI Score}=\left(\mathrm{AST}/\mathrm{ULN}\right)\times 100/\mathrm{PLT}\;\left(10^{9}/L\right);\\ \;\mathrm{FIB}-4\;\text{Index}=\mathrm{Age}\times \mathrm{AST}/\left(\mathrm{PLT}\times \surd \mathrm{ALT}\right). \end{array}$$

#### 2.3.4 Immunohistochemistry to Validate B7‐H6 Expression

Liver tissue paraffin sections from cirrhosis patients were dewaxed and hydrated, followed by antigen retrieval using citrate buffer (pH 6.0). Mouse anti‐human B7‐H6 monoclonal antibody (1:200 dilution) was added and incubated overnight at 4°C. The sections were then incubated with horseradish peroxidase‐labeled secondary antibody, followed by DAB staining and hematoxylin counterstaining. The sections were observed under a microscope, and the staining intensity (0–3) and percentage of positive cells (0–4) were scored to calculate the immunohistochemical score (IHC score).

### 2.4 Statistical Analysis

Statistical analyses were performed using SPSS 26.0 and GraphPad Prism 9.5 software. For continuous variables, normality was first tested, and normally distributed data were expressed as mean ± standard deviation (x̄ ± s), with group comparisons made using one‐way ANOVA. For non‐normally distributed data, values were expressed as median (interquartile range) [M (P25, P75)], and comparisons were performed using the Kruskal‐Wallis H test. Correlation analysis was conducted using Spearman's rank correlation. A *p*‐value of < 0.05 was considered statistically significant. The diagnostic efficacy was evaluated using receiver operating characteristic (ROC) curves to calculate the area under the curve (AUC), sensitivity, and specificity. ROC curve comparisons were made using the DeLong test. To assess the diagnostic efficacy of the combined indicators, a binary logistic regression model based on sB7‐H6 and the traditional non‐invasive marker FIB‐4 was constructed:

$$\text{Logit}\left(P\right)=\beta_{0}+\beta_{1}\times \mathrm{sB}7-\text{H6}+\beta_{2}\times \mathrm{FIB}-4.$$

ROC curves were plotted based on predicted probabilities, and the optimal cutoff value was determined using the Youden index. Variance inflation factor (VIF) testing was conducted to exclude multicollinearity (all VIF < 5). Internal validation was performed using the Bootstrap resampling method (1000 iterations) to assess model stability.

## 3 Results

### 3.1 Baseline Clinical Characteristics of Study Participants

The baseline clinical characteristics of participants in each group are shown in Table 1. There were no significant differences between groups in terms of gender distribution, HBeAg positivity rate, or HBV‐DNA viral load (all *p* > 0.05). However, the age of patients in the LC F4 group was significantly higher than that of the CHB S2‐S3 and HC groups (*p* < 0.05). As liver disease progressed, liver function and hematological markers worsened progressively. ALT, AST, and TBil levels were significantly higher in all patient groups compared to the healthy control group (all *p* < 0.05). Non‐invasive fibrosis biomarkers (APRI, FIB‐4, HA, and LN) showed a strong correlation with the severity of histological findings, with their values increasing significantly from the HC group to the LC F4 group (all *p* < 0.001).

**TABLE 1:** Comparison of clinical characteristics among study groups based on liver histology.

Indicator	Healthy control group (HC) (*n* = 42)	CHB S0–S1 group (*n* = 40)	CHB S2–S3 group (*n* = 37)	LC F4 group (*n* = 82)	*p*
Demographics					
Age (years)	48.5 ± 10.2	46.8 ± 11.5	50.1 ± 9.8	53.2 ± 8.7#&*	< 0.05
Male (*n*, %)	22 (52.4%)	23 (57.5%)	20 (54.1%)	47 (57.3%)	> 0.05
Virological markers					
HBeAg Positive (*n*, %)	—	18 (45.0%)	16 (43.2%)	35 (42.7%)	> 0.05
HBV‐DNA (log_10_ IU/mL)	—	5.2 (3.8, 6.5)	5.5 (4.1, 6.8)	4.9 (3.5, 6.3)	> 0.05
Routine serum markers					
ALT (U/L)	22 (18, 28)	45 (32, 78)Δ	86 (55, 120)Δ#	65 (38, 95)Δ	< 0.001
AST (U/L)	24 (20, 30)	38 (30, 55)Δ	75 (48, 110)Δ#	88 (59, 135)Δ#	< 0.001
TBil (μmol/L)	12.5 (9.8, 15.3)	14.0 (10.5, 18.2)	16.8 (12.0, 22.5)Δ#	28.5 (18.4, 45.0)Δ*#&	< 0.001
Albumin (g/L)	45.2 ± 3.5	42.8 ± 4.1	39.5 ± 3.8Δ#	34.1 ± 5.2Δ*#&	< 0.001
Blood routine					
Platelets (×10^9^/L)	245 ± 55	210 ± 60Δ	155 ± 50Δ#	85 ± 35Δ*#&	< 0.001
White blood cells (×10^9^/L)	6.5 ± 1.5	6.0 ± 1.6	5.3 ± 1.4Δ#	4.5 ± 1.3Δ*#	< 0.001
Liver fibrosis serum models					
APRI	0.3 (0.2, 0.4)	0.6 (0.4, 0.9)Δ	1.4 (0.8, 2.1)Δ#	2.8 (1.6, 4.5)Δ*#&	< 0.001
FIB‐4	1.0 (0.7, 1.3)	1.4 (0.9, 1.9)Δ	2.8 (1.8, 3.9)Δ#	5.5 (3.5, 8.2)Δ*#&	< 0.001
Liver fibrosis four indicators					
HA (ng/mL)	45 (32, 60)	75 (55, 105)Δ	145 (100, 220)Δ#	380 (250, 560)Δ*#&	< 0.001
LN (ng/mL)	95 (78, 115)	120 (95, 150)Δ	180 (135, 250)Δ#	320 (230, 450)Δ*#&	< 0.001
PIII (ng/mL)	13 (11, 18)	11 (8, 14)	14 (10, 22)Δ#	20 (14, 28)Δ*#&	< 0.001
IV‐C (ng/mL)	21 (16, 27)	30 (23, 38)Δ	55 (32, 75)Δ#	76 (35, 122)Δ*#&	< 0.001
sB7‐H6 (pg/mL)	125 (90, 165)	180 (135, 230)Δ	320 (240, 450)Δ#	610 (420, 850)Δ#&*	< 0.001

*Note:* Data are presented as mean ± SD, median (interquartile range), or *n* (%), as appropriate. Overall comparisons among the four groups were performed using one‐way ANOVA, Kruskal–Wallis test, or *χ* ^2^ test, as appropriate. Pairwise comparisons were performed with Bonferroni correction. Δ indicates *p* < 0.05 compared with the HC group; # indicates *p* < 0.05 compared with the CHB S0–S1 group; & indicates *p* < 0.05 compared with the CHB S2–S3 group; * indicates *p* < 0.05 compared with the LC F4 group.

Abbreviations: IV‐C, type IV collagen; ALT, alanine aminotransferase; APRI, aspartate aminotransferase‐to‐platelet ratio index; AST, aspartate aminotransferase; CHB, chronic hepatitis B; FIB‐4, fibrosis‐4 index; HA, hyaluronic acid; HC, healthy control; LC, liver cirrhosis; LN, laminin; PIII, procollagen type III; TBil, total bilirubin.

The levels of sB7‐H6 exhibited a significant and consistent increase in parallel with the progression of liver fibrosis. The serum sB7‐H6 concentration in the CHB S0‐S1 group was significantly higher than in the HC group (180 [135, 230] vs. 125 [90, 165] pg/mL, *p* < 0.05). It rose sharply in the CHB S2‐S3 group (320 [240, 450] pg/mL, *p* < 0.05 compared to CHB S0‐S1) and peaked in the LC F4 group (610 [420, 850] pg/mL, *p* < 0.05 compared to CHB S2‐S3).

### 3.2 Serum sB7‐H6 Levels and Their Relationship With Liver Disease Severity

**FIGURE 1:**
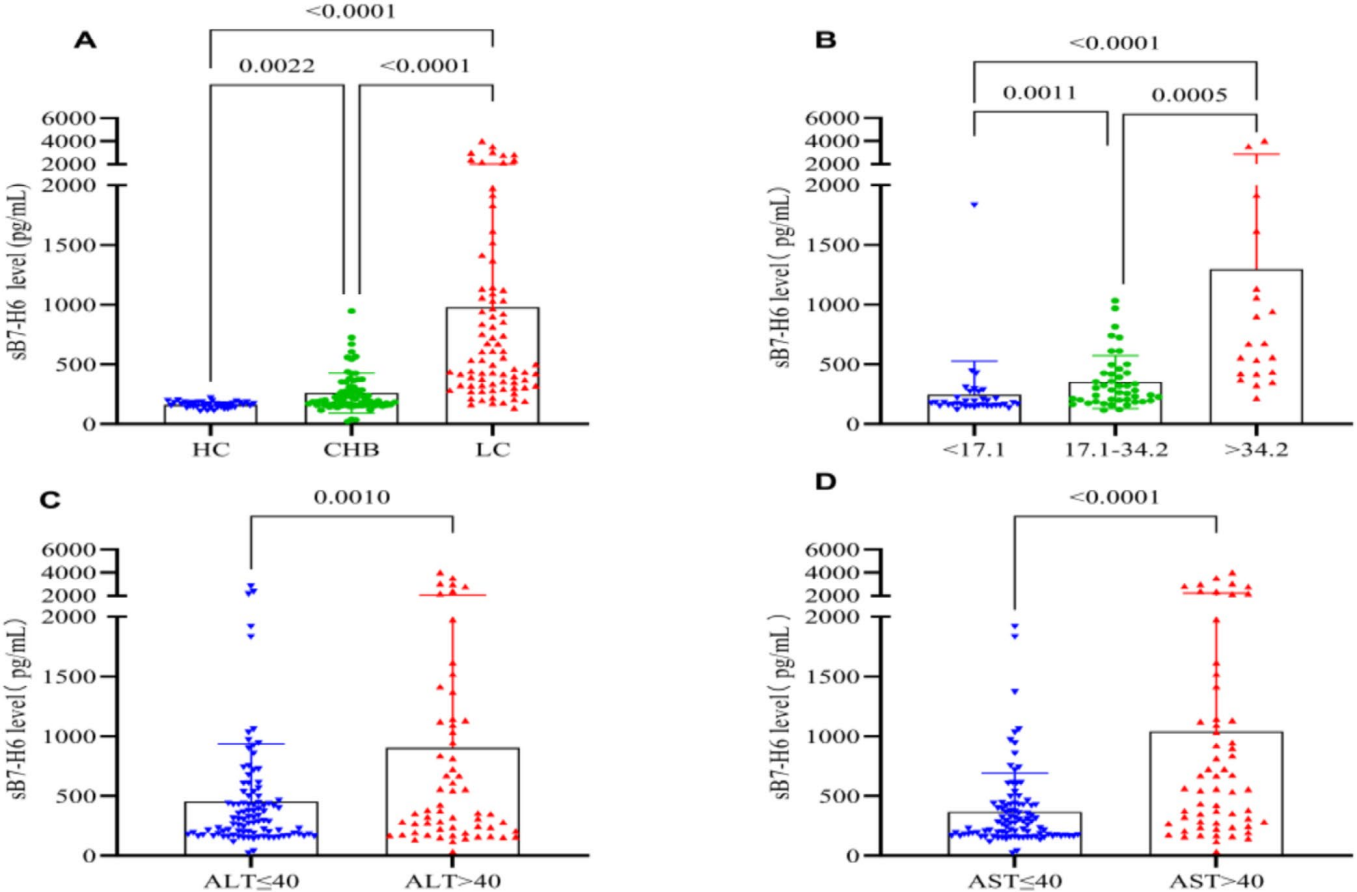
The relationship between serum sB7‐H6 levels and liver disease severity.

Serum sB7‐H6 levels showed significant differences between the groups and increased in a stepwise manner with the progression of liver disease. The sB7‐H6 levels in the LC group were significantly higher than those in the CHB and HC groups (*p* < 0.0001), and the sB7‐H6 levels in the CHB group were also significantly higher than those in the HC group (*p* < 0.0001) (Figure 1A). When grouped according to liver function markers, the sB7‐H6 levels in the elevated TBil, ALT, and AST groups were significantly higher than those in the corresponding normal groups (all *p* < 0.05), suggesting that sB7‐H6 levels are closely related to the degree of liver inflammation and damage (Figure 1B–D).

### 3.3 Serum sB7‐H6 Levels and Their Correlation With Clinical Indicators

#### 3.3.1 Correlation Between sB7‐H6 and Virological Markers

**FIGURE 2:**
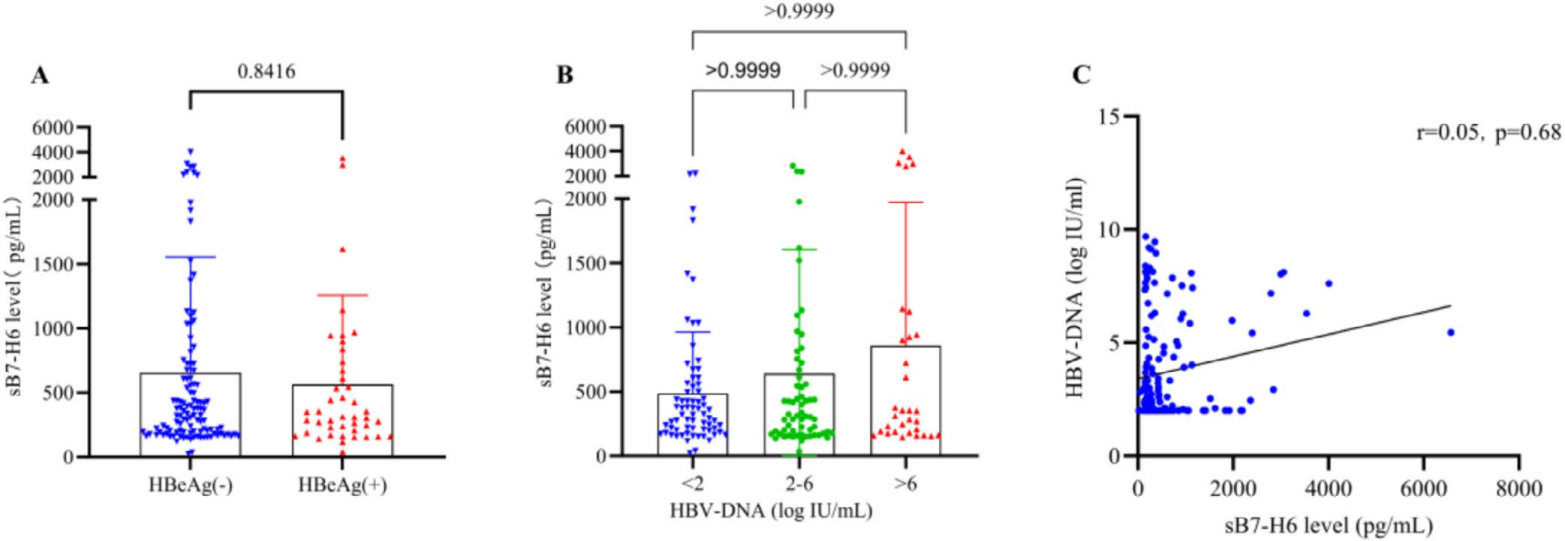
The correlation between sB7‐H6 and virological markers.

To further explore the relationship between sB7‐H6 and HBV replication, we analyzed the correlation between sB7‐H6 levels and HBeAg status as well as HBV‐DNA load. The results showed no significant difference in sB7‐H6 levels between HBeAg‐positive and HBeAg‐negative patients (*p* > 0.05). When patients were grouped based on HBV‐DNA load into low viral load group (< 2 log_10_ IU/mL), medium viral load group (2–5 log_10_ IU/mL), and high viral load group (> 5 log_10_ IU/mL), no significant difference in sB7‐H6 levels was observed among the three groups (*p* > 0.05). Spearman correlation analysis revealed no significant correlation between sB7‐H6 and HBV‐DNA load (*r* = 0.005, *p* = 0.680) (Figure 2).

#### 3.3.2 Correlation Between sB7‐H6 and Liver Injury and Inflammatory Markers

**FIGURE 3:**
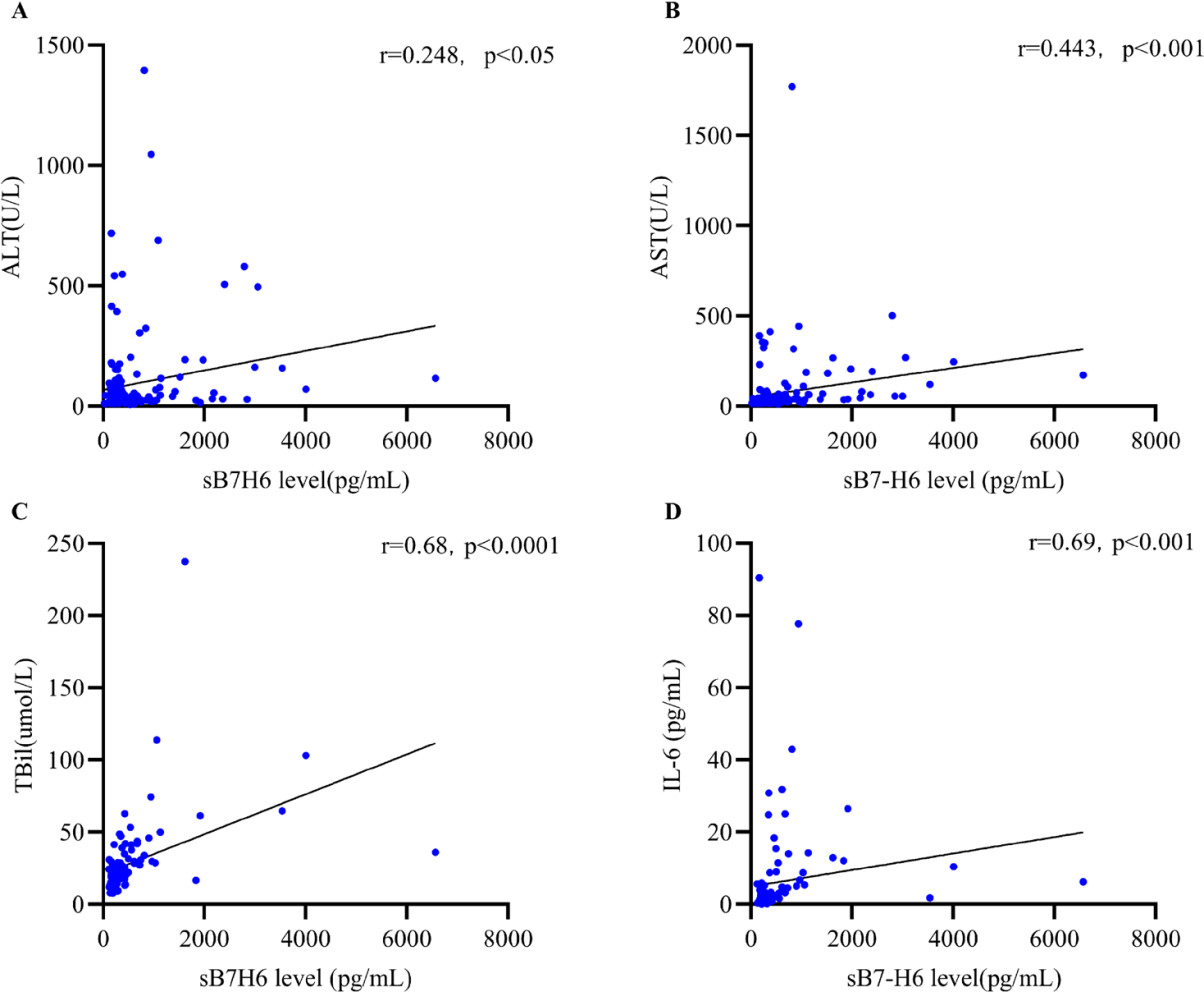
The correlation between sB7‐H6 and liver injury markers in hepatitis B‐related liver disease patients.

Spearman correlation analysis showed that sB7‐H6 levels were significantly positively correlated with ALT (*r* = 0.248, *p* < 0.05), AST (*r* = 0.443, *p* < 0.05), and TBil (*r* = 0.680, *p* < 0.05). Additionally, sB7‐H6 levels were significantly positively correlated with the inflammatory marker IL‐6 (*r* = 0.613, *p* < 0.05). Furthermore, sB7‐H6 levels were also significantly positively correlated with the AST/ALT ratio (De Ritis ratio) (*r* = 0.529, *p* < 0.001), a recognized biochemical indicator of advanced hepatic fibrosis and cirrhosis progression, further supporting the association of sB7‐H6 with progressive hepatic structural and functional deterioration (Figure 3).

#### 3.3.3 Correlation Between sB7‐H6 and Liver Fibrosis Markers

**FIGURE 4:**
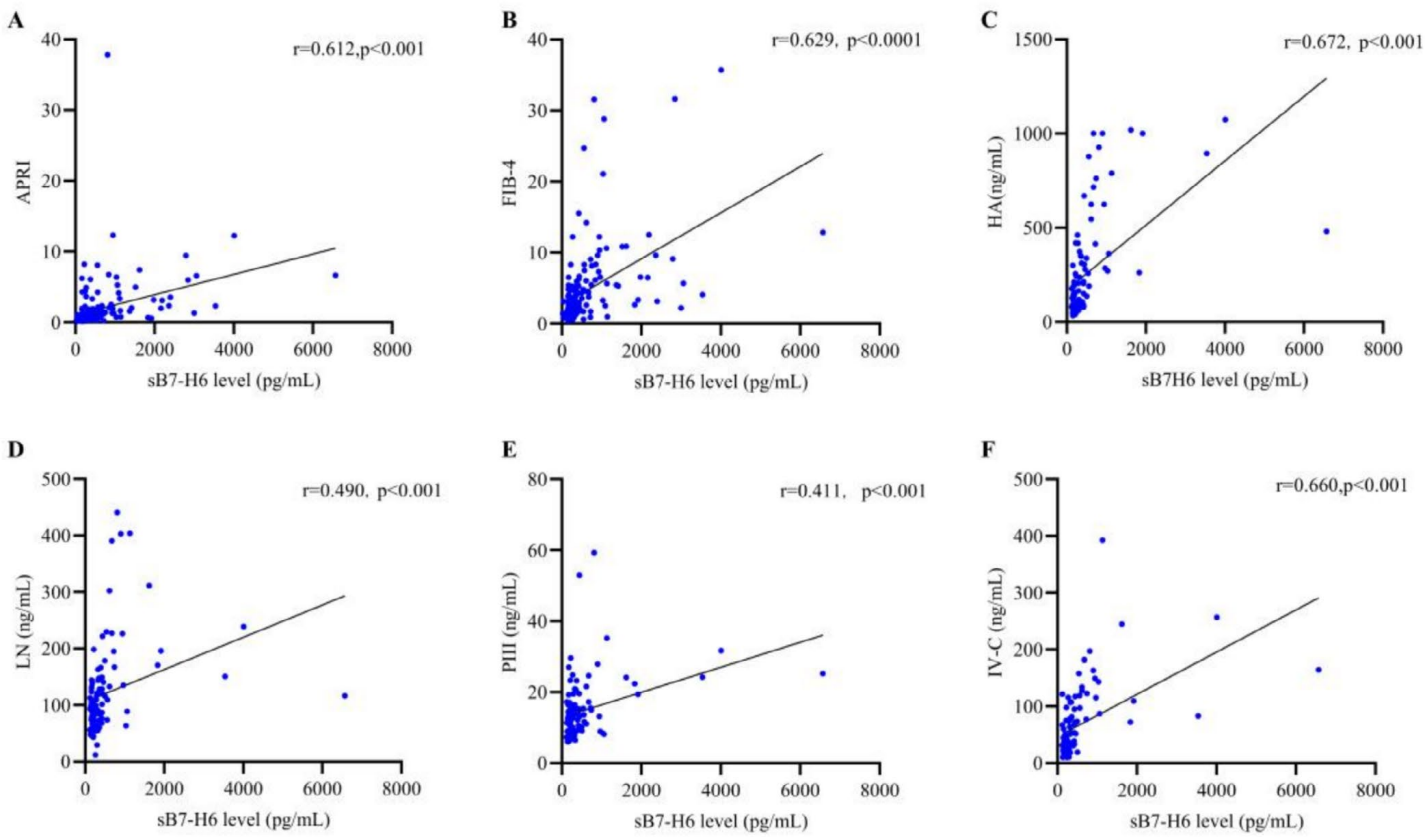
The correlation between sB7‐H6 and liver fibrosis markers.

Spearman correlation analysis showed that sB7‐H6 levels were significantly positively correlated with APRI (*r* = 0.612), FIB‐4 (*r* = 0.629), HA (*r* = 0.672), LN (*r* = 0.490), PIII (*r* = 0.411), and IV‐C (*r* = 0.660) (all *p* < 0.001) (Figure 4).

### 3.4 Diagnostic Value of sB7‐H6 for Liver Cirrhosis

**FIGURE 5:**
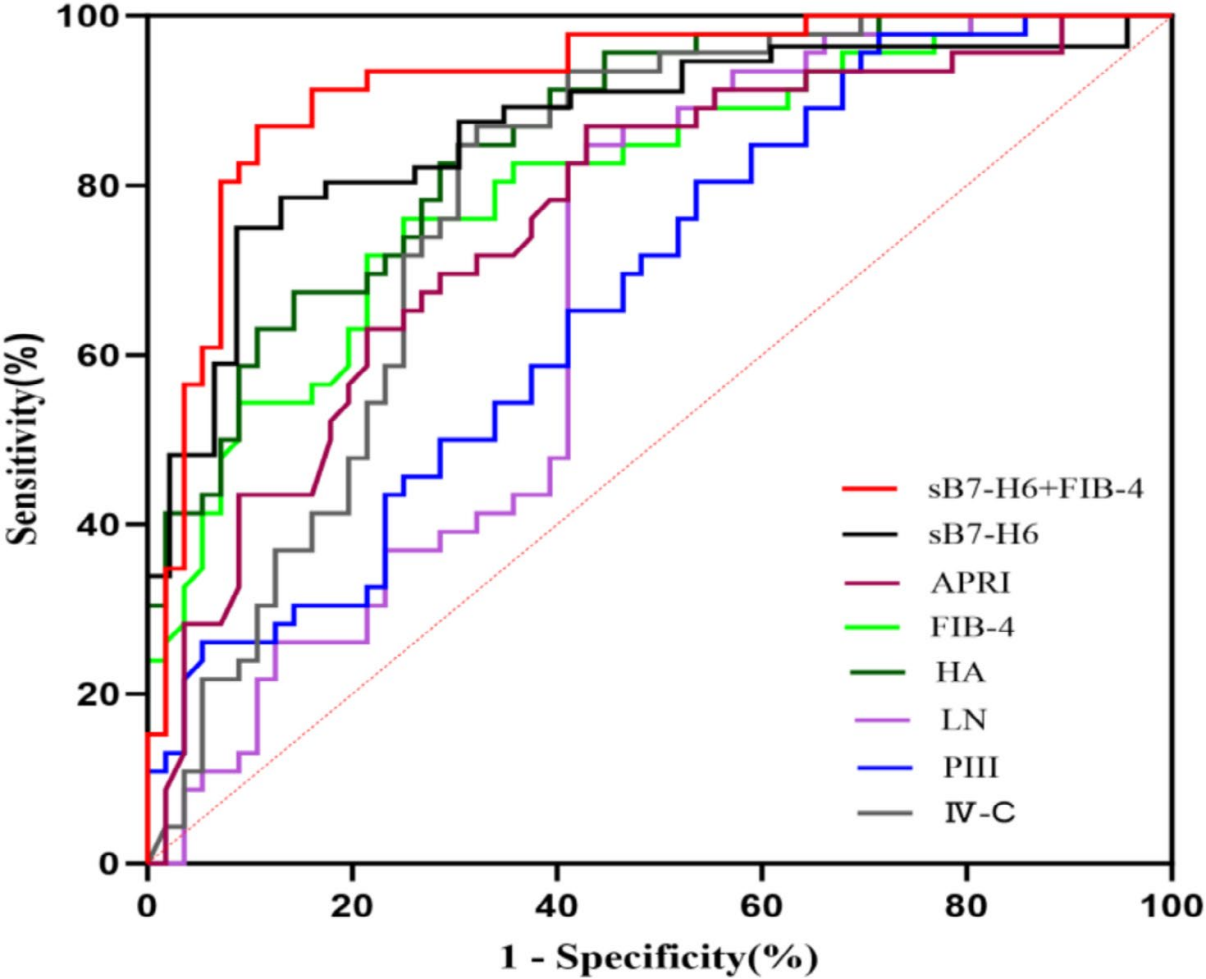
ROC curves for the diagnosis of liver cirrhosis using sB7‐H6, FIB‐4, APRI, and the combined model.

As shown in Table 2 and Figure 5, the AUC for serum sB7‐H6 in diagnosing liver cirrhosis was 0.87 (95% CI: 0.799–0.94), which was superior to the traditional non‐invasive markers, APRI (AUC = 0.72) and FIB‐4 (AUC = 0.75). When sB7‐H6 was combined with FIB‐4 using a binary logistic regression model, the diagnostic performance further improved, with the AUC reaching 0.92 (95% CI: 0.86–0.97), showing a significant increase compared to either single marker (DeLong test, *p* < 0.05). At the optimal cutoff value of 0.48 (predictive probability), the combined model demonstrated a sensitivity of 85.4% and specificity of 88.0%, indicating superior diagnostic accuracy and stability.

**TABLE 2:** Diagnostic performance of various markers for HBV‐related liver cirrhosis.

Indicator	Cut‐off value	Sensitivity (%)	Specificity (%)	AUC (95% CI)	Youden index
sB7‐H6	290	74.5	91.5	0.87 (0.80, 0.94)	0.66
APRI	0.998	56.4	85.1	0.75 (0.65, 0.84)	0.415
FIB‐4	2.5561	74.5	74.5	0.79 (0.70, 0.88)	0.49
HA	160.8	69.1	80.9	0.84 (0.76, 0.91)	0.521
LN	111.1	56.4	83.0	0.66 (0.55, 0.77)	0.394
PIII	14.94	46.0	80.0	0.67 (0.57, 0.77)	0.242
IV‐C	51.29	67.0	85.0	0.78 (0.68, 0.87)	0.524
sB7‐H6‐FIB‐4 Combined	—	85.4	88.0	0.92 (0.86, 0.97)	0.72

## 4 Discussion

This study evaluated the diagnostic value of serum soluble B7‐H6 (sB7‐H6) in hepatitis B virus (HBV)–related cirrhosis. Serum sB7‐H6 levels were significantly elevated in patients with HBV‐related liver disease. Moreover, sB7‐H6 increased stepwise with disease progression. Compared with conventional non‐invasive indices, including APRI and FIB‐4, sB7‐H6 showed superior diagnostic performance for cirrhosis (AUC = 0.87). These findings suggest that sB7‐H6 may serve as an adjunctive serological biomarker.

Serum sB7‐H6 levels were higher in patients with chronic hepatitis B (CHB) and liver cirrhosis (LC) than in healthy controls. The highest levels were observed in the LC group. This pattern is consistent with inducible B7‐H6 expression in inflammatory and tissue injury–associated microenvironments. Previous studies have shown that inflammatory stimuli, such as IL‐1β and TNF‐α, upregulate B7‐H6 expression. The soluble form is generated through ADAM10‐ and ADAM17‐mediated extracellular domain shedding [23, 24]. In HBV‐related acute and chronic liver failure, activation of the B7‐H6–NKp30 axis aggravates hepatocellular injury by inducing IL‐32 expression [27]. In addition, high B7‐H6 expression in hepatocellular carcinoma tissues correlates with inflammatory activity and histological grade [28]. Together, these findings support our results and indicate that elevated sB7‐H6 primarily reflects sustained hepatic immune activation rather than viral replication. Consistently, no significant associations were observed between sB7‐H6 levels and HBV DNA load or HBeAg status.

Correlation analyses showed that sB7‐H6 was positively associated with markers of liver injury, including ALT, AST, and total bilirubin. Significant correlations were also observed with fibrosis‐related indices, such as hyaluronic acid, laminin, procollagen III, and type IV collagen. In addition, sB7‐H6 showed moderate correlations with APRI and FIB‐4. These results suggest that sB7‐H6 reflects both inflammatory activity and fibrotic progression. Importantly, we further identified a significant positive correlation between sB7‐H6 and the AST/ALT ratio (De Ritis ratio) (*r* = 0.529, *p* < 0.001). Because an elevated AST/ALT ratio is widely recognized as a biochemical marker of advanced fibrosis, mitochondrial injury, and reduced hepatocellular reserve, this finding further supports that sB7‐H6 may reflect not only inflammatory activation but also progressive structural and functional deterioration in HBV‐related liver disease. It is therefore unlikely to represent a single pathological process. Previous studies have demonstrated persistent expression of immune checkpoint–related molecules in chronic inflammatory states. Such expression is closely linked to fibrosis severity [13, 29]. Accordingly, sB7‐H6 may represent an integrated indicator of immune activation and immune imbalance during disease progression.

Regarding diagnostic performance, sB7‐H6 achieved an AUC of 0.87 for identifying advanced fibrosis (F4). When combined with FIB‐4, the AUC increased to 0.92. Sensitivity and specificity reached 85.4% and 88.0%, respectively. These findings indicate a clear complementary effect. They also suggest that combining immune‐related biomarkers with structural fibrosis models improves diagnostic accuracy.

sB7‐H6 is not intended to replace transient elastography (TE). Instead, it should be considered a complementary tool. TE reflects liver tissue stiffness but is influenced by obesity, ascites, and operator experience. In contrast, sB7‐H6 is a simple and reproducible serological marker. It may therefore be more suitable for primary healthcare settings or patients unable to undergo imaging‐based evaluation. Combined use of sB7‐H6 and TE allows assessment from both immune and structural perspectives.

A significant positive correlation was observed between sB7‐H6 and IL‐6 levels (*r* = 0.613). This finding supports the hypothesis that inflammatory cytokines drive B7‐H6 expression. Experimental studies have shown that IL‐6 and TNF‐α induce B7‐H6 expression. These cytokines also promote the release of soluble B7‐H6 through ADAM10/17‐mediated cleavage [23, 24]. Therefore, elevated sB7‐H6 likely reflects sustained immune activation rather than transient inflammation. This characteristic may enhance its stability and clinical utility.

## 5 Study Limitations and Future Perspectives

Several limitations should be acknowledged. First, this was a single‐center prospective study. Most participants were recruited from a tertiary hospital. Although consecutive enrollment was used, the cohort may still be biased toward patients with advanced disease. Therefore, the generalizability of the findings to community or primary‐care populations remains uncertain. Second, only internal validation was performed. External validation using independent cohorts is required to confirm robustness and generalizability. Finally, the cellular sources of circulating sB7‐H6 were not investigated. Its precise role in immune–fibrotic regulatory networks also remains unclear. Future studies should address these issues using tissue‐based analyses and functional experiments.

## 6 Study Limitations and Future Perspectives

Several limitations should be acknowledged. First, this was a single‐center prospective study, and most participants were recruited from a tertiary hospital. Although consecutive enrollment was used, the cohort may still be biased toward patients with relatively advanced disease, which may limit the generalizability of the findings to broader community‐based or primary‐care HBV populations. Second, only internal validation was performed; therefore, external validation using independent multicenter cohorts is required to further confirm the robustness, reproducibility, and broader clinical applicability of our findings. Third, the cellular origins of circulating sB7‐H6 were not directly investigated, and its precise mechanistic role within HBV‐associated immune activation, fibrotic remodeling, and immune–fibrotic regulatory networks remains unclear. Future mechanistic studies integrating serum biomarker analysis, liver tissue expression profiling, and immune‐functional experiments are needed to better clarify the biological sources and pathophysiological significance of sB7‐H6 during HBV‐related liver disease progression. In addition, because the present study was primarily designed to evaluate the cross‐sectional diagnostic value of baseline serum sB7‐H6 levels, dynamic longitudinal changes in sB7‐H6 throughout chronic HBV disease progression or during anti‐HBV treatment were not assessed. Therefore, its potential utility as a biomarker for therapeutic efficacy monitoring, fibrosis regression assessment, immune microenvironment remodeling, or long‐term disease surveillance remains undetermined. Future prospective longitudinal studies with serial measurements before and after nucleos(t)ide analogue or other antiviral therapies are warranted to determine whether dynamic sB7‐H6 changes may provide additional value not only for treatment‐response evaluation and disease monitoring, but also for personalized therapeutic stratification and long‐term cirrhosis risk assessment. Collectively, such efforts may help establish sB7‐H6 not only as a complementary diagnostic biomarker, but also as a dynamic and clinically actionable tool across multiple stages of HBV‐related liver disease management.

## 7 Conclusion

This study provides the first systematic evidence supporting the diagnostic value of serum sB7‐H6 in HBV‐related cirrhosis. Serum sB7‐H6 levels were closely associated with liver injury and fibrosis severity. Diagnostic performance was superior to that of conventional serological indices. Combining sB7‐H6 with FIB‐4 further improved diagnostic accuracy. These results highlight the complementary roles of immune activation and structural assessment. sB7‐H6 may therefore serve as a useful adjunctive biomarker for non‐invasive diagnosis and risk stratification in HBV‐related liver disease.

## Author Contributions

Chen Kaiyong and Yang Luxuan contributed equally to this work. Chen Kaiyong: Conception and design of the study, collection and analysis of data, writing of the manuscript, and critical revision of the manuscript for important intellectual content. Yang Luxuan: Data collection, laboratory analysis, statistical analysis, and writing the manuscript. Fu Huaiwu: Assisted in patient recruitment, data analysis, and manuscript revision. Zhu Li: Statistical analysis and interpretation, manuscript revision, and final approval of the manuscript. Cao Lei: Supervision of the study, interpretation of data, and final approval of the manuscript.

## Ethics Statement

This study was approved by the Ethics Committee of the First Affiliated Hospital of Suzhou University (Approval Number: 2021120). All participants provided informed consent. The study adhered to the principles of the Declaration of Helsinki, ensuring the protection of participants' privacy and rights.

## Data Availability

The data that support the findings of this study are available from the corresponding author upon reasonable request.
